# A Case of Solitary Necrotic Nodule Treated with Laparoscopic Hepatectomy: Spontaneous Regression of Hepatocellular Carcinoma?

**DOI:** 10.1155/2013/723781

**Published:** 2013-03-19

**Authors:** Hirokazu Tomishige, Zenichi Morise, Yoshikazu Mizoguchi, Norihiko Kawabe, Hidetoshi Nagata, Hisanori Ohshima, Jin Kawase, Satoshi Arakawa, Rie Yoshida, Masashi Isetani

**Affiliations:** ^1^Department of Surgery, Fujita Health University School of Medicine Banbuntane Houtokukai Hospital, 3-6-10 Otobashi Nakagawaku, Nagoya, Aichi 454-8509, Japan; ^2^Department of Pathology, Fujita Health University School of Medicine Banbuntane Houtokukai Hospital, 3-6-10 Otobashi Nakagawaku, Nagoya, Aichi 454-8509, Japan

## Abstract

Solitary necrotic nodule of the liver is a rare benign lesion with a completely necrotic core and a hyalinized fibrotic capsule containing elastic fibers. The pathogenetic mechanism is still unclear. We here describe a case of SNN, whose central reticulin fibers within the nodule suggest the origin as hepatocellular carcinoma or other hepatocyte-origin tumors, treated with laparoscopic anatomical segmentectomy of the liver. A 76-year-old Japanese female, with no prior medical history and no symptom, visited our hospital with the heterogeneous hypoechoic lesion in the liver segment VI incidentally pointed out in abdominal ultrasonography. Computed tomography with contrast demonstrated a 1.1 cm sized low-density lesion with mild ring enhancement on the rim in the arterial phase. Since the possibility of malignant tumor with necrotic change could not be ruled out, she underwent laparoscopic anatomical segmentectomy of the liver. In the histological examination of the surgical specimen, the liver nodule was necrotic tissue without viable cells and signs of inflammation, which had fibrous capsule and central cystic change and showed trabecular pattern alignment of ghost cells and reticulin fibers orthogonal to the capsule. Also, the findings of chronic hepatitis were observed in the background liver.

## 1. Introduction 

Solitary necrotic nodule (SNN) of the liver is a rare benign lesion first reported in 1983 by Shepherd and Lee, who described four lesions with a completely necrotic core and a hyalinized fibrotic capsule containing elastic fibers [1].

The pathogenetic mechanism is still unclear. In their original study, Shepherd and Lee favored traumatic or infectious etiology [1]. Sundaresan et al. showed the presence of the feeding vessels within the nodule suggesting hemangiomatous origin. They also described central reticulin fibers within the nodule, suggesting the origin as sclerosing hemangioma [2].

We here describe a case of SNN, whose central reticulin fibers within the nodule suggest the origin as hepatocellular carcinoma (HCC) or other hepatocyte-origin tumors, treated with laparoscopic anatomical IV segmentectomy of the liver.

## 2. Case Presentation 

**Figure 1:**
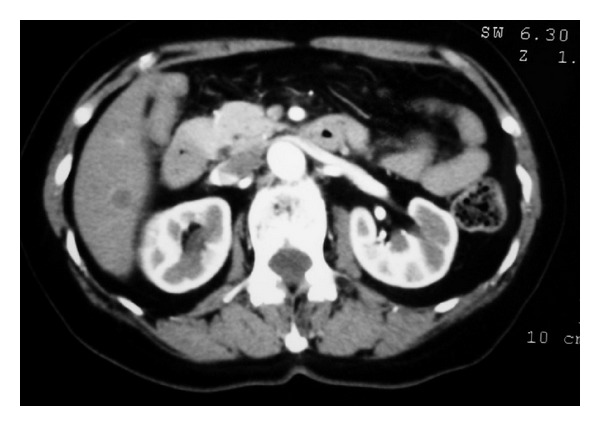
Computed tomography (CT) with contrast demonstrated a 11 mm low-density lesion with mild ring enhancement on the rim in the arterial phase, located in the liver segment VI.

A 76-year-old Japanese female, with no prior medical history and no symptom, visited our hospital with the heterogeneous hypoechoic lesion in the liver segment VI incidentally pointed out in abdominal ultrasonography (US). There were no abnormal findings in her routine laboratory data, including liver function tests, serology profile for hepatitis B or C, and tumor markers including CA 19-9, alpha-Fetoprotein, and CEA. A previous history of alcohol abuse was not documented. Computed tomography (CT) with contrast demonstrated a 1.1 cm sized low-density lesion with mild ring enhancement on the rim in the arterial phase, located in the liver segment VI (Figure 1).

Without any definite diagnosis from clinical imaging, US-guided biopsy was performed. The specimen showed necrotic tissue with trabecular pattern alignment of reticulin fibers in silver stain. The immunohistochemistry examination showed no positive staining with CK7, CK19, CK20, and hepatocyte in the tissue. Since the possibility of hepatocellular carcinoma with necrotic change could not be ruled out, she underwent laparoscopic anatomical IV segmentectomy of the liver.

**Figure 2:**
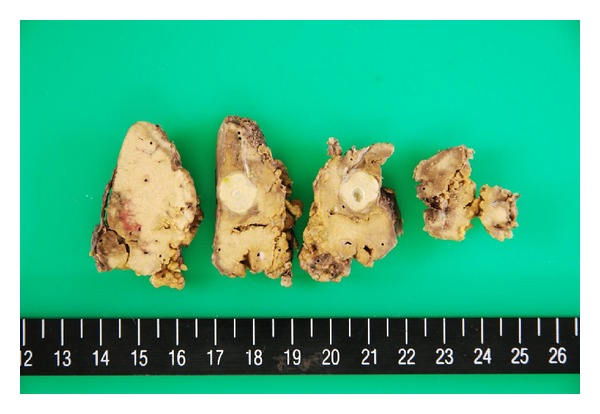
On the cut section of the surgical specimen, a 1.2 × 1.2 cm sized, homogeneous, whitish-yellow-colored nodule with central small cystic area was observed in the liver segment IV. The nodule was well demarcated from surrounding normal liver tissue with the fibrous capsulation.

**Figure 3:**
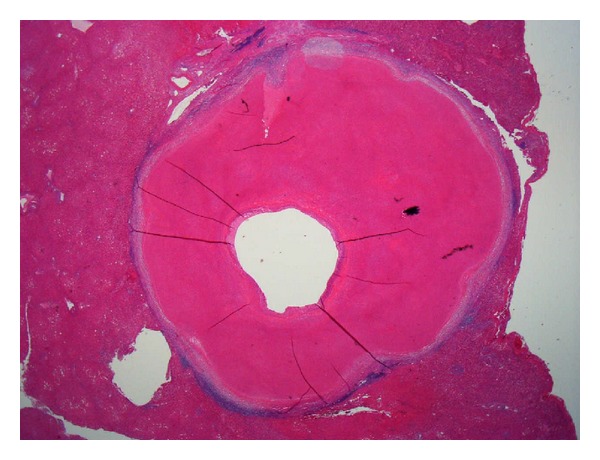
Histologically, the liver nodule was necrotic tissue without viable cells and signs of inflammation, which had fibrous capsule and central cystic change.

**Figure 4:**
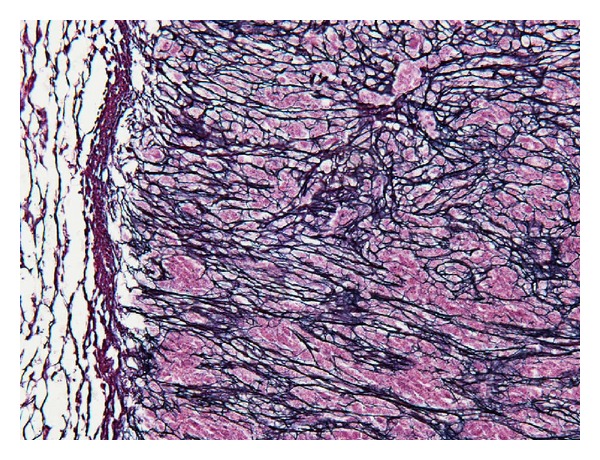
In silver stain, the nodule showed trabecular pattern alignment of ghost cells and reticulin fibers orthogonal to the capsule, which was similar to the hepatocyte-originated neoplasm.

**Figure 5:**
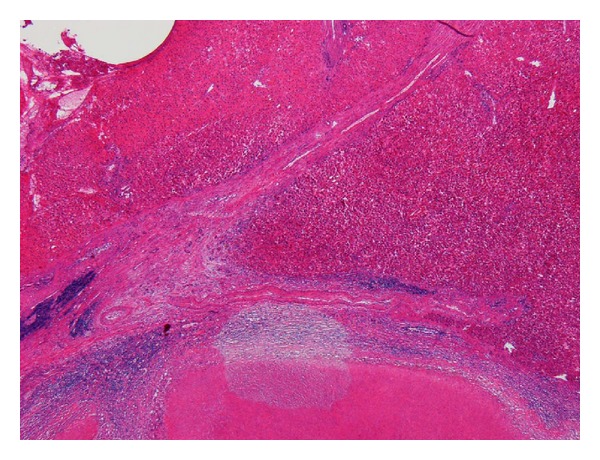
The finding of chronic hepatitis was observed in the background liver.

On the cut section of the surgical specimen, a 1.2 × 1.2 cm sized, homogeneous, whitish-yellow-colored nodule with central small cystic area was observed in the liver segment IV. The nodule was well demarcated from surrounding normal liver tissue with the fibrous capsulation (Figure 2). Histologically, the liver nodule was necrotic tissue without viable cells and signs of inflammation, which had fibrous capsule and central cystic change (Figure 3). In silver stain, the nodule showed trabecular pattern alignment of ghost cells and reticulin fibers orthogonal to the capsule (Figure 4), which was similar to the hepatocyte-originated neoplasm. Also, the findings of chronic hepatitis were observed in the background liver (Figure 5). There were no signs of bacterial or fungal organism and calcification around or inside the lesion. Although the nodule was located next to the peripheral Glissonian pedicle of segment IV, there were no abnormal vessels around or inside the lesion.

Her hospital stay was uneventful and she is well without any signs of recurrence and liver diseases for two and half years after surgery.

## 3. Discussion

SNN is a rare hepatic lesion, pathologically characterized by central amorphous necrotic core, sometimes accompanied with central cystic change, and enclosed by a hyalinized fibrotic capsule [1–4]. In the majority of cases, this condition is clinically silent and often detected incidentally at US examination [5]. Most SNNs are single small lesions and found most commonly under the superficial capsule in the right lobe [2–4]. These characteristics are comparable to the present case. Although SNN is reported to occur in adult males predominantly (68.6% of cases), the present case was in an adult female [4]. 

SNN appears as heterogeneous hypoechoic nodule with unclear margins on US and shows hypodensity lesion with peripheral enhancement when enhanced on CT scan [4]. Differential diagnosis is hard for SNN from intrahepatic cholangiocarcinoma and necrotic metastasis by US and CT scan [6, 7]. Also, percutaneous liver needle biopsy may not be useful for distinguishing SNN from necrotic malignant tumor [8]. In the present case, we also performed surgery under the suspicion of malignant tumor with necrosis, such as HCC.

The etiology of SNN is still unclear. Several pathogenetic hypotheses of SNN are suggested: evolution of hemangioma, lesion of traumatic etiology, and sequelae of previous infection such as parasite [1, 2, 9, 10]. There are previous reports described this entity as a “burnt-out phase” of a variety of lesions and most of them lack specific etiology [11, 12]. However, the alignment of ghost cells and reticulin fibers in the present case is different from previously reported SNN, which origins were suggested as hemangioma or metastasis. Trabecular pattern alignment of ghost cells and reticulin fibers orthogonal to the capsule in the nodule and the findings of chronic hepatitis observed in the background liver may suggest that the nodule is a “burnt-out phase” of hepatocyte-originated neoplasm, such as HCC, although there is no other supporting histological/radiological evidence or definite etiology for hepatitis. On the other hand, there had been reports of spontaneous regression of HCC previously [13, 14]. The cause of the regression is suggested as tumor hypoxia or a systemic inflammatory response [15]. Although there are no evident findings of ischemia (hypoxia) or inflammation in the present lesion, it is possible that an SNN is occurred from spontaneous regression of HCC.

Furthermore, laparoscopic hepatectomy for this lesion with the difficulty of diagnosis should be useful, since it is usually located near the surface of the liver and easy to resect laparoscopically. Even anatomical resection of segment IV for the possibility of HCC in the present case had been performed safely.
